# Social characteristics and social benefit use among premenopausal breast cancer survivors in Denmark: a population-based cohort study

**DOI:** 10.1007/s11764-024-01598-z

**Published:** 2024-04-22

**Authors:** Cathrine F. Hjorth, Julie A. Schmidt, Dóra K. Farkas, Deirdre Cronin-Fenton

**Affiliations:** Department of Clinical Epidemiology, Department of Clinical Medicine, Aarhus University and Aarhus University Hospital, Olof Palmes Allé 43-45, 8200 Aarhus N, Denmark

**Keywords:** Socioeconomic position, Breast neoplasms, Social benefits, Educational status, Cohabitation, Cohort study

## Abstract

**Purpose:**

In 2020, one million women aged < 55 years were diagnosed with breast cancer globally. The impact of breast cancer and its treatments on these women’s ability to work and need for social benefits may differ by social characteristics. We evaluated social benefit use following breast cancer by education and cohabitation.

**Methods:**

We conducted a nationwide population-based cohort study, including women aged 18–55 years diagnosed with stage I-III breast cancer in Denmark during 2002–2011. Statistics Denmark provided information on cohabitation, education, and social benefit use from 1 year pre-diagnosis to 10 years post-diagnosis. We calculated weekly proportions of self-support, unemployment, disability pension, flexi jobs, and sick leave according to education and cohabitation.

**Results:**

Of 5345 women, 81.8% were self-supporting, 4.5% received disability pensions, 1.6% had flexi jobs, 3.6% were on sick leave, and 5.5% were unemployed 1 year pre-diagnosis. Ten years post-diagnosis, the proportions were 69.0%, 13.0%, 10.5%, 3.4%, and 2.0% of 3663 survivors. Disability pensions and flexi jobs increased from 12.1 to 26.4% and 2.8 to 13.5% in women with short education, from 4.1 to 12.8% and 1.8 to 12.2% in women with medium education, and from 0.8 to 6.0% and 0.9 to 6.9% in longer educated. Disability pensions increased more in women living alone (7.8 to 19.9%), than in cohabiting women (3.6 to 11.3%).

**Conclusions:**

Use of social benefits reflecting lost ability to work was highest in less educated women and in women living alone.

**Implications for Cancer Survivors.:**

Awareness of these groups is crucial when tailoring efforts to support work participation in cancer survivors.

**Supplementary Information:**

The online version contains supplementary material available at 10.1007/s11764-024-01598-z.

## Introduction

One-third of all incident breast cancers occur in premenopausal women, and the incidence is increasing in high-income countries [1]. These women are expected to have substantial working years left, owing to advanced breast cancer treatments and improved survival. However, work participation is lower in breast cancer survivors than in their cancer-free counterparts [2–5]. As such, a growing number of breast cancer survivors may lose their ability to work, which is likely to negatively impact their quality of life, mental health, and economy [6, 7].

Premenopausal women tend to have more aggressive tumors and later stage at diagnosis than postmenopausal women. These women are therefore recommended chemotherapy as guideline. Treatment may also include endocrine therapy, radiation therapy, and human epidermal growth factor receptor 2 (HER2) inhibitors. Side effects of these treatments may impact work ability and increase the need for social benefits such as sick leave compensation or disability pensions [2, 8–10].

Lower education level and income are associated with not returning to work after breast cancer either due to sick leave, unemployment, or other circumstances [11]. This has even been seen in studies conducted in welfare systems with universal healthcare access and a high level of social security [2, 3, 5, 12, 13]. Studies document a higher risk of both health-related benefits (e.g., sick leave) and labor market-related benefits (e.g., unemployment benefit) in people with low socioeconomic position [2, 3, 12, 13], but without presenting the total social benefit use in the populations. Two studies evaluated the full extent of social benefit use in women with breast cancer, but one did not account for socioeconomic position [5]. The other study only examined the influence of socioeconomic position on unemployment [3]. Furthermore, many studies included women with metastatic breast cancer, despite the poor prognosis, which is likely to increase social benefit use considerably [2, 3, 5, 14]. Moreover, social support from spouse or partner may be an important factor to regain or maintain the ability to work after breast cancer.

We therefore conducted a nationwide, population-based cohort study of premenopausal women diagnosed with stage I-III breast cancer in Denmark to investigate the total pattern of social benefit use before, during, and after diagnosis, and evaluated the impact of the social characteristics education and cohabitation.

## Methods

### Study design and setting

This cohort study was conducted in Denmark. Danish residents have uniform access to tax-funded health care and are safeguarded economically by the government in case of workforce detachment, sickness, or social issues limiting their labor market contribution [15, 16]. Several programs are implemented to help people return to work including vocational rehabilitation programs, job search assistance, counselling, and guidance. Upon free access to education, students aged ≥ 18 years receive a government-funded student grant each month (the State Education Grant). All social benefit payouts are recorded on a weekly basis and are linkable to other Danish administrative or health registries through a unique personal identifier assigned to all Danish residents upon birth or immigration [17].

### Study population

We nested our cohort in the ProBe CaRe (Predictors of Breast Cancer Recurrence) cohort [18]. The ProBe Care cohort (*n *= 5959) is derived from a nationwide population of premenopausal women diagnosed with stage I-III primary breast cancer in Denmark during 2002–2011, and registered in the Danish Breast Cancer Group (DBCG) clinical database. Since 1977, the DBCG database has recorded all women under 70 years diagnosed with early-stage breast cancer in Denmark [19, 20]. Menopausal status at diagnosis was collected from the DBCG clinical database, but to minimize the risk of misclassification, we restricted our study population to women aged 18–55 years at date of surgery. The ProBe CaRe cohort comprised women who had estrogen receptor (ER)-positive breast cancer who initiated tamoxifen therapy, as well as women with ER-negative breast cancer who did undergo endocrine therapy [18]. For this study, we also restricted to all women who received at least one cycle of chemotherapy ensuring representation of those on guideline treatment regimens. We excluded women who had immigrated to Denmark less than 1 year prior to their breast cancer diagnosis, defined as the date of surgery.

### Education and cohabitation

We ascertained data on the highest achieved level of education at the date of breast cancer diagnosis from the Danish Population’s Education Registry [21]. In accordance with the International Standard Classification of Education 2011 (ISCED 2011) [22], we categorized education level into short (ISCED 0–2), intermediate (ISCED 3–4), and long education (ISCED 5–8). Based on information from the Danish Civil Registration System [17], we categorized women as living alone or as cohabiting if they were married, in a registered partnership or living with a partner in accordance with criteria listed in Supplemental Table S1.

### Social benefits

We obtained records of social benefit payments from the Danish Register for Evaluation of Marginalization (DREAM) from 1 January 2001 to 31 January 2021. The DREAM registry contains validated data on payments of sick leave compensation (long-term), unemployment benefits (part or full time), and other health and social benefits, registered weekly, since mid-1991 [23, 24].

We categorized social benefits into the following groups: self-supporting, unemployment, sick leave, disability pension, flexi job, other health-related benefits, other labor market-related benefits, and a censored group, which included women no longer expected to be part of the workforce due to retirement, recurrence, or other malignancies (Table 1). We considered people as self-supporting if they had no registered benefit in DREAM [25], or if they received the State Educational Grant [26]. Where women received maternity leave benefits during follow-up, we recoded these weeks into the category prior to the maternity leave. Additional details are included in Supplemental Table S3.

**Table 1:** Social benefit groups derived from entries in the DREAM database during 2001–2021

	Types of benefits
Self-supporting	No benefit (proxy for employed or self-supporting) and student grants
Unemployment	Unemployment, incl. part-time unemployment
Sick leave	Long-term sick leave
Disability pension	Disability pension
Flexi job schemes	Scheme supporting people with reduced working capacity to remain in the workforce
Other health-related benefits	Vocational rehabilitation programmes and workability clarification (often prior to granted disability pension)
Other labor market-related benefits	Primarily social security/assistance

Sick leave registered in DREAM captures instances of long-term sick leave, recorded from the first day of illness until the end of the leave. Notably, short-term sick leave episodes, lasting less than 14–30 days (with the specific duration dependent on the calendar year, as outlined in Supplemental Table S4), are not included in the registry.

In Denmark, disability pensions are awarded to people with a permanent, substantial reduced work ability [27]. People with reduced work ability may be entitled to flexi jobs if they do not qualify for disability pensions. Flexi jobs are schemes where those employed have reduced working hours, to accommodate their work ability. The employer is entitled to a partial wage subsidy [28].

### Covariates

From the DBCG clinical database [18], we collected information on age at diagnosis, date of surgery, ER status, HER2 status, grade (for ductal or lobular tumors) and number of positive lymph nodes, and tumor size to assign TNM (Tumor Node Metastasis) stage [29]. We collected information on type of surgery (mastectomy or lumpectomy including intention to treat radiation therapy) and chemotherapy (at least one cycle received), and dates of new primary cancers and breast cancer recurrences, adhering to DBCG’s definition as loco-regional, distant, or contralateral breast cancer [30]. From the Danish National Patient Registry, we collected information on comorbidities [31]. We summarized the number of comorbidities (except breast cancer) using the Charlson Comorbidity Index (Supplemental Table S1) [32]. We also collected dates of emigration and death from Statistics Denmark.

### Statistical analyses

We described the study cohort using standard descriptive statistics. We graphically illustrated weekly proportions of receipt of social benefits from 1 year before breast cancer diagnosis and up to 10 years after. Women were censored—and hence excluded from the denominator—at date of retirement, breast cancer recurrence, new primary cancers, emigration, or death. We calculated absolute differences (percentage points, pp) in social benefit use 1 year pre-diagnosis and 10 years post-diagnosis to evaluate the influence of breast cancer. In addition, we stratified analyses by cohabitation and education status to examine social gradients. Further, we illustrated social benefit trajectories via a Sankey diagram using the same social benefit groups and an additional group extracting retirements from the censored group. All statistical analyses were conducted using SAS 9.4 (SAS Institute, Cary, NC).

#### Additional analyses

We repeated all the above analyses without censoring on recurrence and new primary cancers, to encompass the total social benefit use related to breast cancer and subsequent cancers.

## Results

In total, 5375 women in the ProBeCaRe cohort were treated with at least one cycle of chemotherapy and aged 18–55 years. After excluding 30 women due to immigration to Denmark less than 1 year before date of breast cancer diagnosis, the study cohort included 5345 women. Cohort characteristics are presented in Table 2. Most of the breast cancers were stage II (55.0%), ER + (77.1%), HER2 − (59.3%), and among patients with ductal or lobular carcinoma, 44.5% were grade 2.

**Table 2:** Cohort characteristics

	*N*	%
Total	5345	100
Age at diagnosis		
18–35	359	6.7
35–44	1988	37.2
45–55	2998	56.1
Estrogen receptor status		
ER −	1223	22.9
ER +	4122	77.1
HER2 status		
Negative	3170	59.3
Positive	879	16.4
Missing	1296	24.2
Stage^a^		
Stage I	1292	24.2
Stage II	2939	55.0
Stage III	1086	20.3
Missing	28	0.5
Histological grade^b^		
Grade 1	825	15.4
Grade 2	2381	44.5
Grade 3	1700	31.8
Not graded	375	7.0
Missing	64	1.2
Surgery type^c^		
Mastectomy	2434	45.5
Lumpectomy incl. intention-to-treat radiotherapy	≤ 291	-
Missing	≤ 5	-
Comorbidity Index Score		
0	4739	88.7
1–2	500	9.4
3 or more	106	2.0
Cohabitation		
Cohabiting	4223	79.0
Living alone	1106	20.7
Missing	16	0.3
Educational level		
Short	1063	19.9
Medium	2198	41.1
Long	2025	37.9
Missing	59	1.1
Ethnicity		
Danish extraction	4987	93.3
Immigrant or decendant	351	6.6
Missing	7	0.1

^a^Derived from tumor size and lymph node status

^b^Ductal and lobular tumors. Other tumors were not graded

^c^In accordance with Danish data protection rules, data from cell sizes < 5 individuals and cells permitting back calculation are reported in aggregate

### Overall social benefit use

**Fig. 1:**
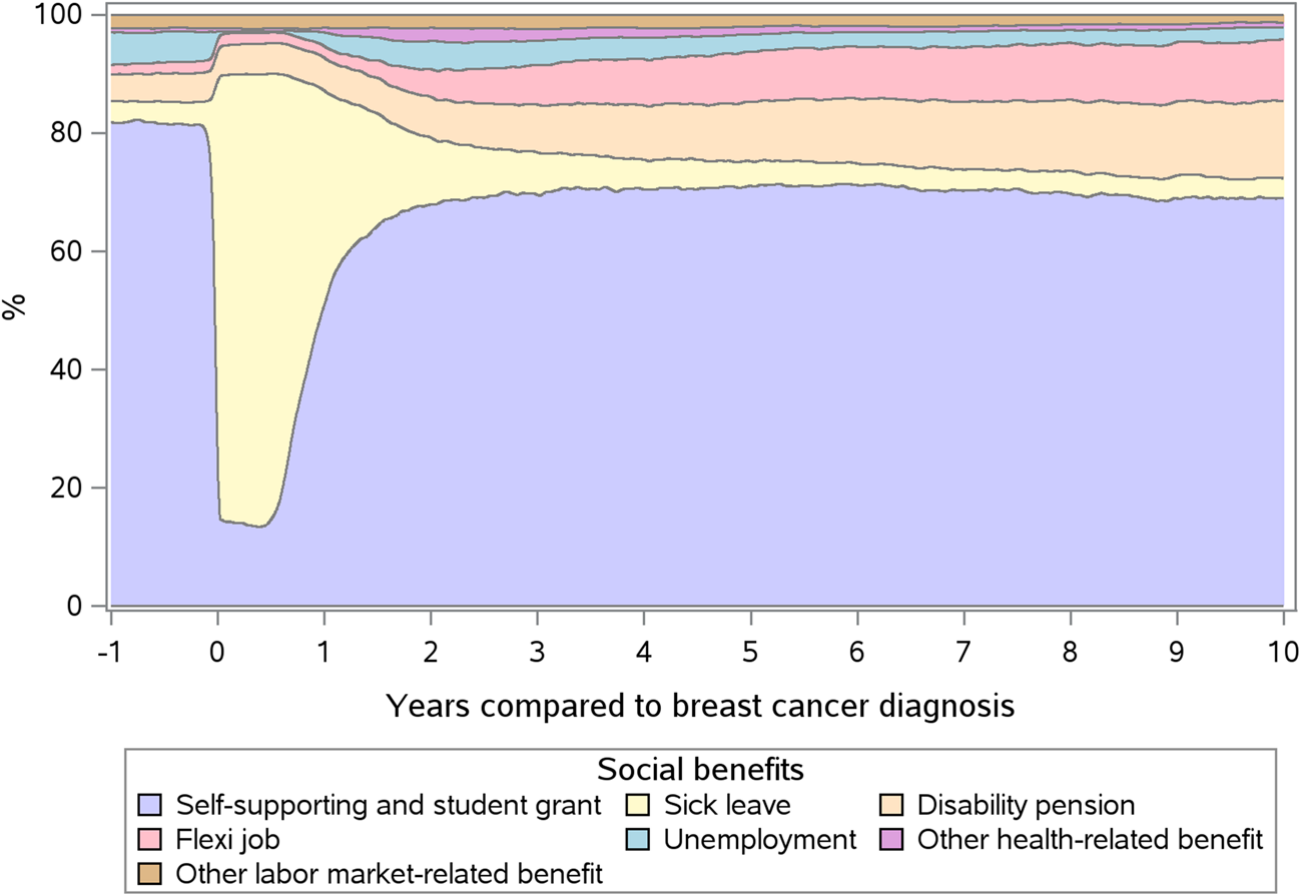
Weekly proportions of social benefit use from 1 year before to 10 years after breast cancer diagnosis in premenopausal breast cancer survivors

The impact of breast cancer diagnosis on social benefit use was evident from a few weeks before diagnosis with a pronounced drop in self-supportiveness and an equivalent increase in sick leave at diagnosis (Fig. 1 and Table 3). One year after, half of the women were self-supporting, increasing to 68.1% after 2 years and remaining stable thereafter. In the week of diagnosis, 73.0% of women were on sick leave, which increased slightly in the first 6 months after diagnosis—the period when the women were undergoing chemotherapy, and dropped steadily afterwards reaching the pre-diagnostic level. Pre-diagnosis, 4.5% received a disability pension and 1.6% had flexi jobs. These proportions increased during follow-up to 13.0% and 10.5% at 10 years post-diagnosis, amounting to a 9 pp increase in both types of benefits. The proportion of unemployment decreased from 5.5% before diagnosis to 2.0%, 10 years after diagnosis (− 4 pp).

**Table 3:** Pre- and post-diagnostic social benefit use by education and cohabitation status, and absolute differences over time

	One year pre-diagnosis (− 1)		Week of diagnosis (0)		Two years post-diagnosis (+ 2)		Five years post-diagnosis (+ 5)		Ten years post-diagnosis (+ 10)		Absolute difference, − 1 and + 10 years
	*N* %		*N* %		*N* %		*N* %		*N* %		pp
**Self-support**	**4367**	**81.8**	**885**	**16.6**	**3432**	**68.1**	**3241**	**71.1**	**2527**	**69.0**	** − 13**
Cohabiting	3551	84.1	719	17.0	2823	70.8	2689	74.0	2088	71.3	− 13
Living alone	809	73.1	163	14.7	599	57.7	543	59.9	428	59.3	− 14
Short education	708	66.6	120	11.3	531	53.5	475	53.1	370	52.0	− 15
Medium education	1784	81.3	356	16.2	1372	66.0	1314	70.2	1005	66.8	− 14
Long education	1838	90.8	398	19.7	1502	78.6	1427	82.2	1134	81.1	− 10
**Disability pension**	**240**	**4.5**	**266**	**5.0**	**348**	**6.9**	**457**	**10.0**	**477**	**13.0**	**9**
Cohabiting	154	3.6	175	4.1	236	5.9	317	8.7	331	11.3	8
Living alone	86	7.8	91	8.2	112	10.8	140	15.4	144	19.9	12
Short education	129	12.1	136	12.8	174	17.5	190	21.3	188	26.4	14
Medium education	90	4.1	101	4.6	122	5.9	181	9.7	193	12.8	9
Long education	16	0.8	23	1.1	46	2.4	77	4.4	84	6.0	5
**Flexi job**	**88**	**1.6**	**104**	**1.9**	**236**	**4.7**	**388**	**8.5**	**383**	**10.5**	**9**
Cohabiting	60	1.4	73	1.7	175	4.4	293	8.1	295	10.1	9
Living alone	28	2.5	31	2.8	61	5.9	95	10.5	88	12.2	10
Short education	30	2.8	34	3.2	73	7.4	105	11.7	96	13.5	11
Medium education	40	1.8	44	2.0	105	5.1	181	9.7	183	12.2	10
Long education	18	0.9	26	1.3	54	2.8	95	5.5	96	6.9	6
**Sick leave**	**193**	**3.6**	**3899**	**73.0**	**564**	**11.2**	**185**	**4.1**	**125**	**3.4**	**0**
Cohabiting	145	3.4	3146	74.5	429	10.8	145	4.0	109	3.7	0
Living alone	48	4.3	746	67.5	134	12.9	38	4.2	16	2.2	− 2
Short education	49	4.6	698	65.7	93	9.4	41	4.6	14	2.0	− 3
Medium education	94	4.3	1626	74.0	260	12.5	81	4.3	50	3.3	− 1
Long education	47	2.3	1544	76.3	201	10.5	60	3.5	58	4.1	2
**Unemployment**	**293**	**5.5**	**37**	**0.7**	**237**	**4.7**	**134**	**2.9**	**72**	**2.0**	− **4**
Cohabiting	226	5.4	29	0.7	183	4.6	101	2.8	57	1.9	− 3
Living alone	67	6.1	8	0.7	54	5.2	33	3.6	15	2.1	− 4
Short education	84	7.9	8	0.8	56	5.6	35	3.9	19	2.7	− 5
Medium education	131	6.0	17	0.8	113	5.4	54	2.9	39	2.6	− 3
Long education	76	3.8	12	0.6	68	3.6	44	2.5	12	0.9	− 3

Danish data protection rules do not allow publishing cells with < 5 individuals. To prevent back-calculation of such cells, other cells need to be masked/not reported as well. Therefore, other health-related and labor market-related benefits are not reported in these analyses, and percentages do not add up to 100

**Fig. 2:**
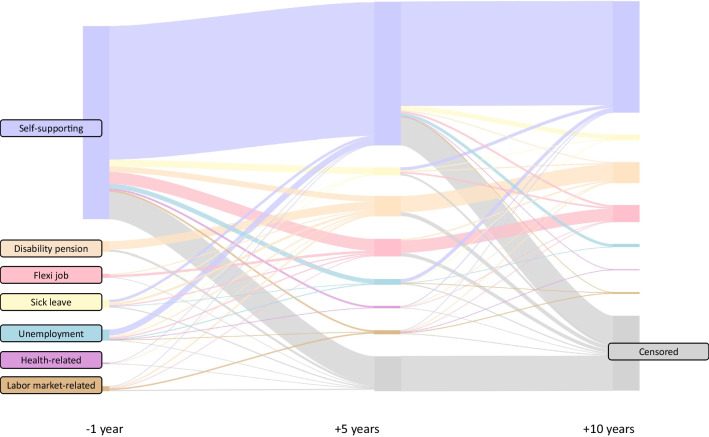
Social benefit trajectories from 1 year before breast cancer diagnosis to 10 years after in premenopausal breast cancer survivors. The nodes and flows represent percentages of women at risk. In accordance with Danish data protection rules, trajectory groups with ≤ 5 individuals were deleted. The censored group included women experiencing other malignancies, breast cancer recurrence, immigration, or death

As seen from Fig. 2, among those who were self-supporting before diagnosis, 54.3% maintained their self-supportiveness, 3.5% retired, 3.4% were granted disability pensions, and 6.3% had flexi jobs 10 years after their diagnosis. A total of 20.6% of the self-supporting women were censored due to immigration, new primary cancers, breast cancer recurrence, or death. Among those unemployed before diagnosis, 33.1% were self-supporting 10 years after diagnosis, and 10.2% and 11.3% had disability pensions and flexi jobs, respectively. One-quarter of those on sick leave before diagnosis received disability pensions 10 years after. All proportions related to Fig. 2 are available in Supplemental Table S5.

### Cohabitation

**Fig. 3:**
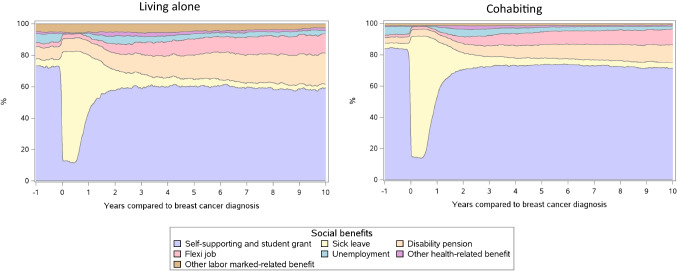
Weekly proportions of social benefit use from 1 year before breast cancer diagnosis to 10 years after in premenopausal breast cancer survivors by cohabitation status

Among women living alone, 73.1% were self-supporting 1 year before diagnosis, 7.8% were on disability pensions, 2.5% had flexi jobs, 4.3% were on sick leave, and 6.1% were unemployed (Fig. 3 and Table 3). Among cohabiting women, these proportions were 84.1%, 3.6%, 1.4%, 3.4%, and 5.4%, respectively.

In the week of diagnosis, the proportion of those self-supporting dropped to 17.0% and 14.7% in cohabiting women and in women living alone, respectively. Two years after, 70.8% and 57.7%, respectively, were self-supporting, which was stable thereafter. At 10 years, 19.9% were on disability pensions and 12.2% in flexi jobs among women living alone. In cohabiting women, this was 11.3% and 10.1%. The absolute increase in the proportion on disability pensions was higher among those living alone, than in cohabiting women (12 pp vs. 8 pp).

### Education level

**Fig. 4:**
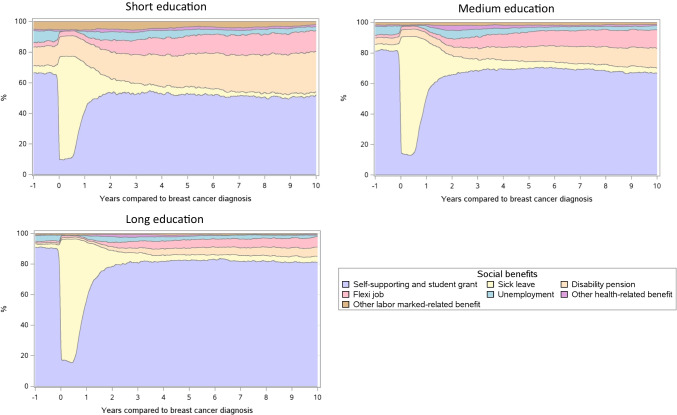
Weekly proportions of social benefit use from 1 year before breast cancer diagnosis to 10 years after in premenopausal breast cancer survivors with short, medium, and long education

The proportions of women who were self-supporting 1 year before breast cancer diagnosis were 66.6%, 81.3%, and 90.8% in those with short, medium, and long education, respectively (Fig. 4 and Table 3). These proportions dropped to 11.3%, 16.2%, and 19.7% in the week of breast cancer diagnosis. Two years after, the groups reached almost steady state of self-supportiveness of 53.5%, 66.0%, and 78.6%, respectively. The absolute differences indicated a social gradient in self-supportiveness according to education level as the decreases were larger among women with short or medium education (− 15 pp and − 14 pp) than in women with long education (− 10 pp).

We also found a negative education gradient in disability pension and flexi job with larger increases in the proportions among those with shorter education. The proportion of patients who received disability pensions increased from 12.1 to 26.4% (14 pp) in those with short education, from 4.1 to 12.8% (9 pp) in those with medium education, and from 0.8 to 6.0% (5 pp) in those with long education. Likewise, the proportion of women on flexi jobs increased from 2.8 to 13.5% (11 pp) in those with short education, from 1.8 to 12.2% (10 pp) in those with medium education, and from 0.9 to 6.9% (6 pp) in those with long education. The proportion of women on sick leave and unemployment were similar across education levels.

### Results of additional analyses

The proportions found in the analyses including social benefits granted after a recurrence or a new primary cancer during follow-up were similar to those from the main analyses (Supplemental Figure S1 and Table S6).

## Discussion

In this nationwide, population-based cohort of premenopausal women, we observed distinct social inequality in work participation and social benefit use after breast cancer. Compared with cohabiting women, disability pensions were more frequent in women living alone, and those with short education were more likely to be granted disability pensions and flexi jobs after their breast cancer than women with higher education.

In the USA, 27–40% of those employed at diagnosis were unemployed 3–5 years after breast cancer diagnosis [33–35]. One study found that women with low income had higher unemployment risk, but found no association with education [34]. Research in the USA suggests that health insurance status may affect work participation after breast cancer. A study including 708 women aged ≤ 45 years with early-stage breast cancer found that after 18 months, 56% and 16% were in full-time and part-time employment, respectively. Only 2.4% were disabled; 35% of the women were fearful of changing jobs due to the risk of losing their health insurance, and 26% experienced insurance-related problems post-diagnosis, e.g., increasing costs or insurance denial [36]. These issues may also be related to socioeconomic position. Other factors of importance in the USA include ethnicity and race, with lower rates of returning to work and higher proportions of sick leave in black women than in white women [37–39]. During the study period, immigrant/descendant women had markedly higher use of disability pensions in Denmark than non-immigrants/descendants, mainly due to mental health issues [40]. Still, fewer than 7% of our study population were immigrants or descendants, precluding any further analyses of social benefits according to ethnicity.

Our observed education gradient in self-supportiveness aligns with a study by Heinesen et al. [14] on Danish women with breast cancer diagnosed during 2000–2004. They found that the probability of losing employment increased by 9 pp among women with short education and by 5 pp among those with long education [14]. Heinesen et al. may have underestimated the education gradient as they excluded women who permanently left the workforce before breast cancer, died, or had a subsequent cancer diagnosis within 3 years of breast cancer diagnosis, which is more prevalent in women with short education [41]. Still, job type (blue vs. white-collar) explained some of the gradient; blue-collar workers are more likely to have short education level and physically demanding jobs.

Our observed drop in unemployment after breast cancer diagnosis echoes findings from a Dutch study [5]. Research suggests that unemployed people are more likely to leave the workforce permanently after cancer [14]. However, we found that one-third of the unemployed women became self-supporting, though some entered flexi jobs or disability pensions.

A Swedish study by Kvillemo et al. [42] reported that 34.7% of breast cancer survivors were on sick leave 2 years after diagnosis, which was considerably higher than in our study (11.3%). They found no changes in the proportion on disability pensions. Yet, Kvillemo and colleagues captured short-term sick leave (12.1%), which may explain some of the differences as short-term sick leave is not captured in DREAM. Still, diverse social policies across countries may also account for the observed differences. In Sweden, disability pensions can be part-time granted alongside sick leave [42], somewhat similar to the flexi job scheme in Denmark.

In Denmark, disability pensions and the flexi job schemes are reserved for those deemed unable to return to the work force, or with substantially reduced work ability. Our findings may therefore indicate debilitating breast cancer sequelae affecting the ability to work in women with low socioeconomic position and women living alone. This inequality is likely attributable to a complex interplay of various factors. Compared with people with long education, people with short education are more than twice as likely to have comorbidities [43]. Comorbidities are associated with increased risk of adverse events after cancer, which may impede a return to work after cancer*.* Further, a study by Dalton et al. [44] among 13,059 cancer survivors in Denmark found that women with breast cancer were as likely to be referred to rehabilitation after cancer regardless of their education level. However, those with long education were more likely to attend than those with medium or short education [44]. We note that Dalton et al. did not find any differences in referral to rehabilitation programs or attendance by cohabitation. Cohabiting women may have greater social support during diagnosis and treatment. Accordingly, these women may be more likely to maintain or regain their work ability, than women living alone.

### Strengths and limitations

The strengths of this study include the detailed description of social benefit use in breast cancer survivors over an 11-year period, and the nationwide administrative and health care registries with complete follow-up. Registration of information on social benefits in DREAM has high validity compared with self-reported and employer-reported data [23, 24]. In fact, some benefits may be most valid when collected in the registries, as these go directly to the employer and not the individual [24]. Some women categorized as self-supporting may not have been working. Instead, these women may have been supported by their spouse or savings—a situation that might be more likely in cohabiting women and those with long education. However, the definition of self-supportiveness has been validated against self-report with a positive predictive value of 98% [24].

This study also has some limitations. Social security benefit policies continuously change, impacting the prevalence of specific benefits over time. Due to a change in the Disability Pension Act in 2013 tightening the criteria (Supplemental Table S4), contemporary cancer survivors may have a lower prevalence of disability pensions and higher prevalence of flexi jobs if diagnosed at a young age. Moreover, flexi jobs became temporary, and how this affects contemporary breast cancer survivors is yet unknown. Nonetheless, inequality in work ability after breast cancer may not change with these legislations. We note that our pre- and post-diagnostic comparison is somewhat compromised due to a decreasing denominator over time, especially in women living alone and/or those with shorter education as the rates of breast cancer recurrence and mortality are higher in these groups [41]. However, the inequality was similar when including women with recurrences in the analyses. Directly comparing our results to the general population would provide valuable context and enhance the external validity of our study, allowing us to better understand the relative uptake of social benefits. Unfortunately, we did not have access to a general population cohort in the current study.

### Perspectives

Our long follow-up and detailed description of social benefit use patterns may inform timing of intervention strategies and target populations among breast cancer survivors. A recent systematic review found a lack of evidence on effective interventions to support work participation after cancer among unemployed or work-disabled cancer survivors. Most studies pointed towards multicomponent interventions (e.g., job search assistance and vocational training) like the standard social efforts provided in Denmark [45]. Literature also suggests that job type and employer’s willingness to offer flexible working schedules (as distinct from flexi jobs) can support a return to work after illness [46, 47]. A flexible work schedule could include working or staying home when mentally or physically challenged. The positive effect of being offered flexible working schedules has mainly been seen in cancer survivors with manual jobs and in people with short education [48]. This may be because people with long education are less likely to have manual jobs and already possess jobs with a high level of flexibility [48].

## Conclusions

Despite Denmark’s uniform access to healthcare and guaranteed income security, we found a negative education gradient in disability pensions and flexi jobs among breast cancer survivors. Moreover, women living alone were more likely to receive disability pensions. These findings suggest that the likelihood of losing the ability to work after breast cancer was inversely associated with education level and cohabitation status. As such, women with shorter education and women living alone may benefit from programs aimed at supporting work force participation after breast cancer. Mechanistic insights may be needed to design such programs.

## Supplementary Information

Below is the link to the electronic supplementary material.

Supplementary file1 (DOCX 349 KB)

## Data Availability

The data used for the current study derive from nationwide, population-based administrative and medical registries, linked anonymously using the Civil registration a personalized-identifier. The data are available from Statistics Denmark, DBCG, and the corresponding author. Restrictions apply to the availability of these data, which were used under license for this study. Data are available from the authors upon reasonable request with the permission of Statistics Denmark, DBCG, The Danish Health Authorities, The Danish Data Protection Agency, and The Central Jutland Region Committee on Health Research Ethics.
