# Supplementary material for: Social characteristics and social benefit use among premenopausal breast cancer survivors in Denmark: a population-based cohort study
Source: J Cancer Surviv. 2024 Apr 22;19(6):1835–45. doi: 10.1007/s11764-024-01598-z (PMC12546285; doi:10.1007/s11764-024-01598-z)
Supplement: Supplementary file 1 — Supplementary file1 (DOCX 349 KB) [file 11764_2024_1598_MOESM1_ESM.docx]

**Supplemental material**

**Socioeconomic position and social benefit use among premenopausal breast cancer survivors in Denmark**

**Content**

[Table S1. Algorithm for Charlson Comorbidity Index revision 2](#_Toc146280433)

[Table S2. Coding table 3](#_Toc146280434)

[Table S3. Employment status categorized by weekly entries in DREAM 4](#_Toc146280435)

[Table S4. Characteristics and changes of legislations in Denmark 5](#_Toc146280436)

[Figure S1. Weekly proportions of social benefit use from one year before breast cancer diagnosis to 10 years after in premenopausal breast cancer survivors, without censoring on recurrence and other malignancies 6](#_Toc146280437)

[Table S5. Social benefit trajectories from one year before diagnosis to 10 years after 7](#_Toc146280438)

[Table S6. Social benefit use 10 years after breast cancer and the absolute differences with reference to one year before breast cancer, without censoring on recurrence and other malignancies. 8](#_Toc146280439)

[References 9](#_Toc146280440)

## Table S1. Algorithm for Charlson Comorbidity Index containing International Classification of Diseases, 8^th^ and 10^th^ revision

|  | **Diseases** | **ICD-8** | **ICD-10** | **Score** |
| --- | --- | --- | --- | --- |
| 1 | Myocardial infarction | 410 | I21;I22;I23 | 1 |
| 2 | Congestive heart failure | 427.09; 427.10; 427.11; 427.19;  428.99; 782.49 | I50; I11.0; I13.0; I13.2 | 1 |
| 3 | Peripheral vascular disease | 440; 441; 442; 443; 444; 445 | I70; I71; I72; I73; I74; I77 | 1 |
| 4 | Cerebrovascular disease | 430-438 | I60-I69; G45; G46 | 1 |
| 5 | Dementia | 290.09-290.19; 293.09 | F00-F03; F05.1; G30 | 1 |
| 6 | Chronic pulmonary disease | 490-493; 515-518 | J40-J47; J60-J67; J68.4; J70.1; J70.3; J84.1; J92.0; J96.1; J98.2; J98.3 | 1 |
| 7 | Connective tissue disease | 712; 716; 734; 446; 135.99 | M05; M06; M08; M09; M30; M31; M32; M33; M34; M35; M36; D86 | 1 |
| 8 | Ulcer disease | 530.91; 530.98; 531-534 | K22.1; K25-K28 | 1 |
| 9 | Mild liver disease | 571; 573.01; 573.04 | B18; K70.0-K70.3; K70.9; K71; K73; K74; K76.0 | 1 |
| 10 | Diabetes type1  Diabetes type2 | 249.00; 249.06; 249.07; 249.09  250.00; 250.06; 250.07; 250.09 | E10.0, E10.1; E10.9  E11.0; E11.1; E11.9 | 1 |
| 11 | Hemiplegia | 344 | G81; G82 | 2 |
| 12 | Moderate to severe renal disease | 403; 404; 580-583; 584; 590.09;  593.19; 753.10-753.19; 792 | I12; I13; N00-N05; N07; N11; N14; N17-N19; Q61 | 2 |
| 13 | Diabetes with end-organ damage  type1  type2 | 249.01-249.05; 249.08  250.01-250.05; 250.08 | E10.2-E10.8  E11.2-E11.8 | 2 |
| 14 | Any tumor (except breast cancer) | 140-194 (excluding 174) | C00-C75 (excluding C50) | 2 |
| 15 | Leukemia | 204-207 | C91-C95 | 2 |
| 16 | Lymphoma | 200-203; 275.59 | C81-C85; C88; C90; C96 | 2 |
| 17 | Moderate to severe liver disease | 070.00; 070.02; 070.04; 070.06;  070.08; 573.00; 456.00-456.09 | B15.0; B16.0; B16.2; B19.0; K70.4; K72; K76.6; I85 | 3 |
| 18 | Metastatic solid tumor | 195-198; 199 | C76-C80 | 6 |
| 19 | AIDS | 079.83 | B21-B24 | 6 |

The Danish National Patient Registry covers all Danish hospitals and has registered data on all non-psychiatric inpatient admissions since 1977 and outpatient and emergency room visits since 1995 [1].

## Table S2. Coding table

| **Covariate** | **Definition** | **Data source** |
| --- | --- | --- |
| Age group | Age at date of diagnosis | DBCG |
| ER status | Tumors assessed prior to 1. July 2010 were deemed ER– if ER expression was <10%, and ER+ if ≥10%, which was limited to a 1% cut off afterwards | DBCG |
| HER2 status | Categorized as positive or negative | DBCG |
| Stage | According to the TNM staging system [2] | DBCG |
| Anaplastic grade | Grade 1–3 assigned ductal and lobular tumors. Others were not graded | DBCG |
| Surgery type | Mastectomy or lumpectomy incl. ITT radiation therapy | DBCG |
| Recurrence | Date of diagnosed recurrence | DBCG |
| Other malignancy | Date of other diagnoses of malignancies | DBCG |
| Comorbidities | Summarized according to the Charlson Comorbidity Index [3] | The Danish National Patient Registry. The algorithm is inserted in Table S1 |
| Cohabitation | Women with or without a partner | Statistics Denmark. Women were considered as having a partner if fulfilling one of following criteria 1) being married, 2) in a registered partnership, 3) live on same address as person with common child, 4) if living with one person of the opposite sex and age difference is less than 15 years, no children, and living at the same address. The variable is updated annually in November |
| Education | Short education: ISCED 0–2 ~ <10 years of education  Medium education: ISCED 3–4 ~ 10 to 14 years of education  Long education: ISCED 5–8 ~ ≥15 years of education | Danish Population’s Education Registry [4] |
| Social benefits |  | The DREAM database. See categorization in Table S3 |

Abbreviations: DBCG= Danish Breast Cancer Group, DREAM= Danish Register for Evaluation of Marginalization, ER= Estrogen receptor, HER2= Human epidermal growth factor receptor 2, ISCED= International Standard Classification of Education, ITT= Intention-to-treat, TNM= Tumor node metastasis

## Table S3. Employment status categorized by weekly entries in The Danish Register for Evaluation of Marginalization (DREAM)

| **Entries** | **Description** | **Status** |
| --- | --- | --- |
| No entry | No transfer payment | Self-supporting |
| 521 | Adult trainee |  |
| 651 652 661 662 794 | State Education Fund grants |  |
| 122 123 | Vacation payment used during employment |  |
| 996 | Retirement age, but not retired |  |
| 890 891-899 | Sick leave benefits | Sick leave |
| 774 | Sick leave from flexi job |  |
| 781 | Light duty during disability pension | Disability pension |
| 783 784 797 793 | Disability pension |  |
| 761-762 769 771-774 779 782 796 | Flexi job | Flexi job |
| 622 | Early retirement after flexi job |  |
| 740 743-748 | Unemployed awaiting flexi job |  |
| 111 114 | Unemployment benefit all week | Unemployed |
| 112 113 115 | Unemployment benefit part time |  |
| 121 124-126 | Vacation payment from unemployment |  |
| 211-219 221 222 224 225 231 232 297-299 511 522 541 722 759 | Unemployment benefit during special efforts e.g. job training or supervision |  |
| 750 753-758 760 763-768 791 792 | Rehabilitation | Other health-related benefits |
| 810 813-819 784 | Vocational rehabilitation program |  |
| 785 870 873-879 | Workability clarification |  |
| 411 413 | Leave-of-absence schemes | Other labor market-related benefits |
| 160 163-169 | Ready for employment benefit |  |
| 710-719 | Social benefit, immigrants |  |
| 704-709 | Immigration benefit during job training |  |
| 140-149 151 414 700 703 720-729 732 742 751 752 | Education assistance, not health related |  |
| 130-139 141-142 152-153 730-739 741 | Social assistance, not health related |  |
| 611, 621 | Post-employment retirement | Censored |
| 997 | Not resident in Denmark |  |
| 998 | Retirement |  |
| 999 | Death |  |
| 881, 412 | Maternity leave | Categorized according to the week prior to code entry |
| 795 | Benefits due to sick child |  |

## Table S4. Characteristics and changes of legislations in Denmark

| ***Sick leave***  Short term sick leave is not included in DREAM, as this period is paid by the employer (*i.e.* the employee receives regular salary), hence, no social benefits are granted. The duration of this period was increased during follow up, starting with 14 days until 2007, 15 days until June 2008, 21 days until 2011 and hereafter 30 days.  When entering long term sick leave the employer are compensated economically, which is registered in DREAM. The preceding weeks of employer paid sick leave are then added to DREAM.  People with chronic illness are counted sick from their first day of absence. |
| --- |
| ***Disability pension and flexi jobs***  People granted disability pension has been through a resource scheme concluding a substantial reduction in work capacity precluding them from work, including flexi jobs. To be granted disability pension the person should be a Danish citizen and have lived in Denmark for least 3-10 years, depending on age [5]. A structural reform of the Disability Pension Act in 2013 changed the criteria, so that people under age 40 cannot (with few exemptions) be awarded disability pension and the criteria for getting disability pension were generally tightened [6]. Instead, people were enrolled vocational training programs and/or awarded flexi jobs. In a flexi job, the person works for a substantially reduced hours per week in a job tailored the persons work capacity. Before 2013, flexi jobs were mainly permanent, after 2013, they became temporary. |
| ***Unemployment***  In Denmark, members of an unemployment insurance fund can receive unemployment compensation full time or part time for a given period. A reform in May 2010 gradually reduced the period in which a person can receive unemployment benefits from 4 years to 2 years. Before the reform, 26 weeks of employment was required to quality for unemployment benefits. This was raised to 52 weeks [7]. |

## Figure S1. Weekly proportions of social benefit use from one year before breast cancer diagnosis to 10 years after in premenopausal breast cancer survivors, without censoring on recurrence and other malignancies

| **All women**  **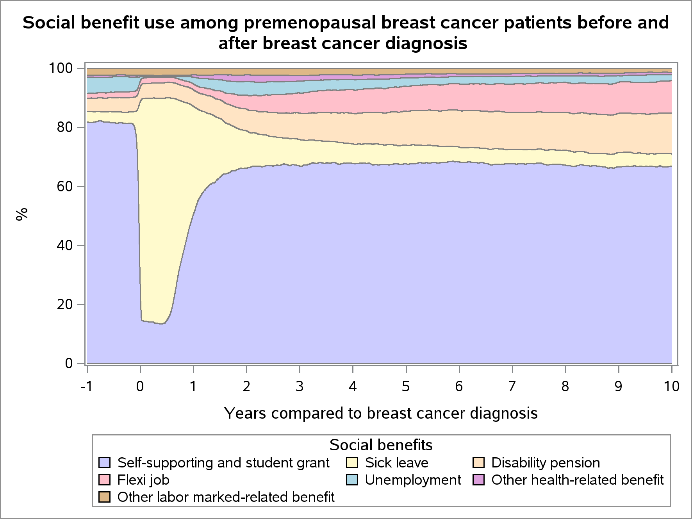** | **Short education**  **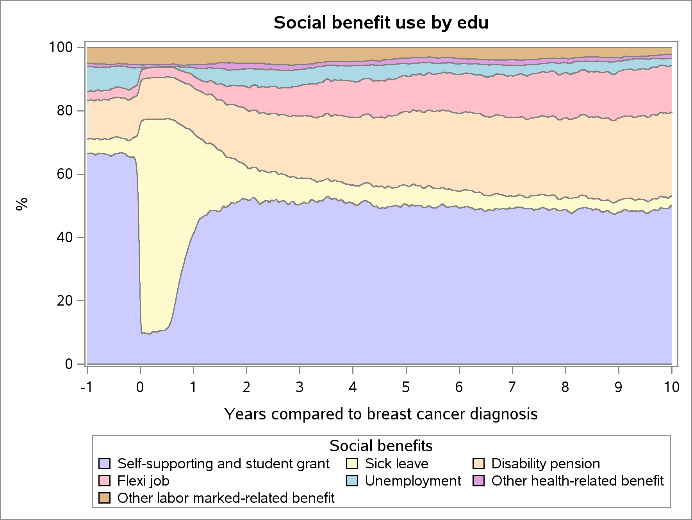** |
| --- | --- |
| **Living alone**  **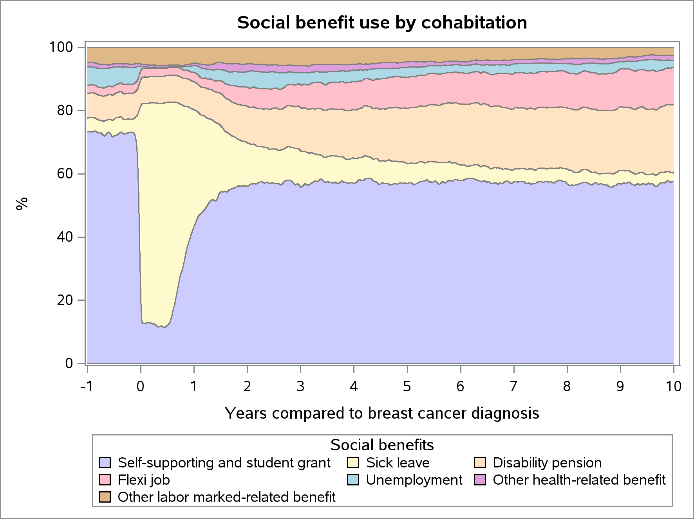** | **Medium education**  **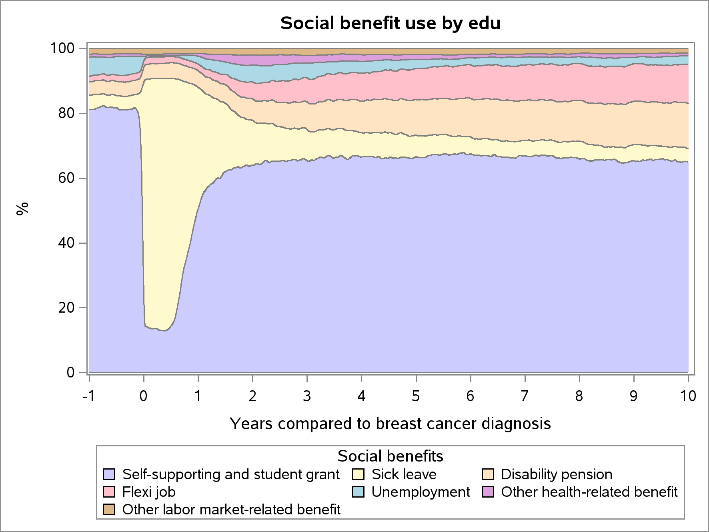** |
| **Cohabiting**  **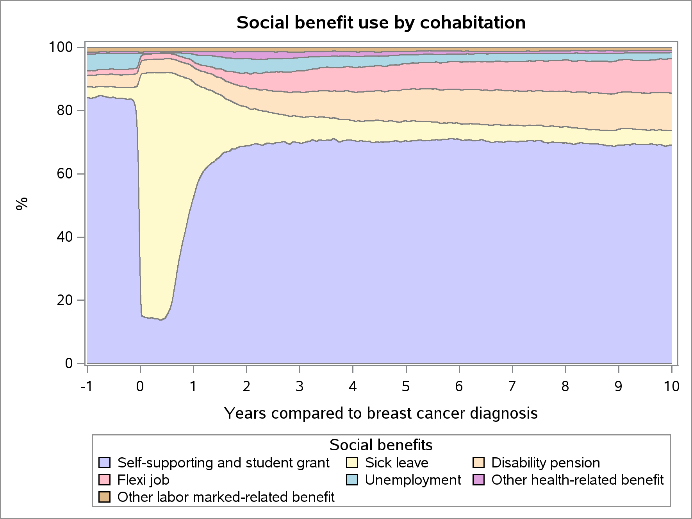** | **Long education**  **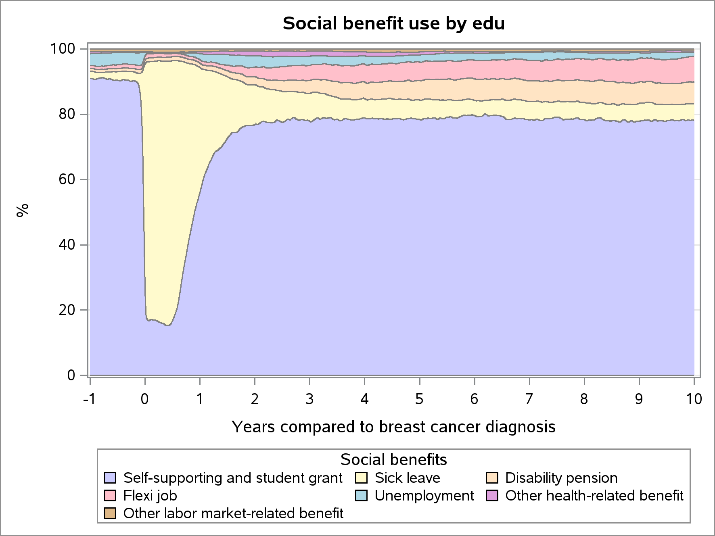** |
| **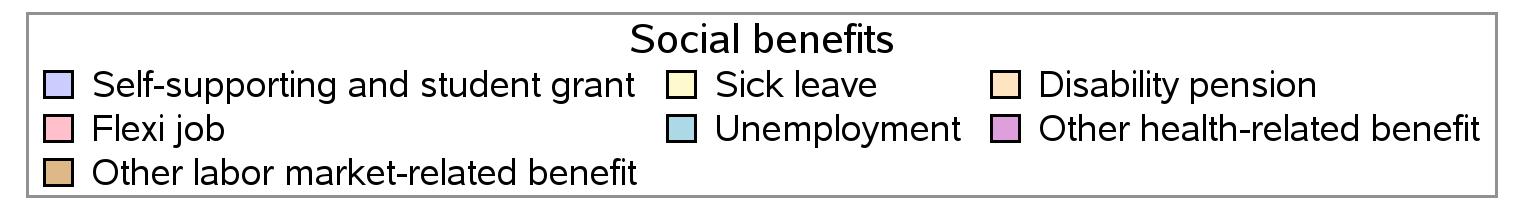** | |

## Table S5. Social benefit trajectories from one year before diagnosis to 10 years after

| **One year pre-diagnosis**  **(-1)** | **Ten years post-diagnosis**  **(+10)** | **Frequency Count** | **Percent** |
| --- | --- | --- | --- |
| Self-supporting | Self-supporting | 2373 | 54.3 |
|  | Sick leave | 105 | 2.4 |
|  | Disability pension | 182 | 4.2 |
|  | Flexi job | 276 | 6.3 |
|  | Unemployed | 59 | 1.4 |
|  | Other health-related | 12 | 0.3 |
|  | Other labor market-related | 22 | 0.5 |
|  | Censored | 899 | 20.6 |
|  | Unknown | 284 | 6.5 |
|  | Retired | 155 | 3.5 |
| Sick leave | Self-supporting | 37 | 19.2 |
|  | Disability pension | 47 | 24.4 |
|  | Flexi job | 25 | 13.0 |
|  | Other health-related | 6 | 3.1 |
|  | Censored | 40 | 20.7 |
|  | Unknown | 25 | 13.0 |
| Disability pension | Disability pension | 156 | 65.0 |
|  | Censored | 61 | 25.4 |
|  | Unknown | 23 | 9.6 |
| Flexi job | Disability pension | 17 | 19.3 |
|  | Flexi job | 35 | 39.8 |
|  | Censored | 21 | 23.9 |
|  | Unknown | 13 | 14.8 |
| Unemployed | Self-supporting | 97 | 33.1 |
|  | Sick leave | 11 | 3.8 |
|  | Disability pension | 30 | 10.2 |
|  | Flexi job | 33 | 11.3 |
|  | Unemployed | 11 | 3.8 |
|  | Other health-related | 6 | 2.0 |
|  | Other labor market-related | 7 | 2.4 |
|  | Censored | 74 | 25.3 |
|  | Unknown | 15 | 5.1 |
|  | Retired | 9 | 3.1 |
| Other health-related | Self-supporting | 8 | 20.0 |
|  | Disability pension | 8 | 20.0 |
|  | Censored | 14 | 35.0 |
| Other labor market-related | Self-supporting | 8 | 6.7 |
|  | Disability pension | 37 | 31.1 |
|  | Flexi job | 12 | 10.1 |
|  | Other health-related | 7 | 5.9 |
|  | Other labor market-related | 14 | 11.8 |
|  | Censored | 35 | 29.4 |
| Danish data protection rules do not allow publishing cells with <5 individuals. Therefore, some groups are not reported, and percentages may not add up to 100. | | | |

## Table S6. Social benefit use 10 years after breast cancer and the absolute differences with reference to one year before breast cancer, without censoring on recurrence and other malignancies

|  | **Ten years**  **post-diagnosis**  **(+10)** | | **Absolute difference,**  **-1 and +10 years** |
| --- | --- | --- | --- |
|  | N % | | pp |
|  |  |  |  |
| **Self-support** | 2682 | 66.8 | -15 |
|  |  |  |  |
| Cohabiting | 2217 | 69.1 | -15 |
| Living alone | 454 | 57.5 | -16 |
|  |  |  |  |
| Short education | 393 | 50.1 | -16 |
| Medium education | 1064 | 65.1 | -16 |
| Long education | 1206 | 78.1 | -13 |
|  |  |  |  |
| **Disability pension** | 553 | 13.8 | 9 |
|  |  |  |  |
| Cohabiting | 381 | 11.9 | 8 |
| Living alone | 170 | 21.5 | 14 |
|  |  |  |  |
| Short education | 207 | 26.4 | 14 |
| Medium education | 228 | 13.9 | 10 |
| Long education | 105 | 6.8 | 6 |
|  |  |  |  |
| **Flexi job** | 442 | 11.0 | 9 |
|  |  |  |  |
| Cohabiting | 349 | 10.9 | 9 |
| Living alone | 93 | 11.8 | 9 |
|  |  |  |  |
| Short education | 116 | 14.8 | 12 |
| Medium education | 197 | 12.0 | 10 |
| Long education | 121 | 7.8 | 7 |
|  |  |  |  |
| **Sick leave** | 170 | 4.2 | 1 |
|  |  |  |  |
| Cohabiting | 149 | 4.6 | 1 |
| Living alone | 21 | 2.7 | -2 |
|  |  |  |  |
| Short education | 22 | 2.8 | -2 |
| Medium education | 66 | 4.0 | 0 |
| Long education | 79 | 5.1 | 3 |
|  |  |  |  |

Danish data protection rules do not allow publishing cells with <5 individuals. To prevent back-calculation of such cells, other cells need to be masked/not reported as well. Therefore, unemployment are not reported in these analyses.

Abbreviations: pp = percentage points, N = numbers

## References

1. Schmidt M, Schmidt SAJ, Sandegaard JL, Ehrenstein V, Pedersen L, Sørensen HT. The Danish National Patient Registry: a review of content, data quality, and research potential. Clin Epidemiol. 2015;7:449–90.

2. Amin MB, American Joint Committee on Cancer, American Cancer Society, editors. AJCC cancer staging manual. Eight edition / editor-in-chief, Mahul B. Amin, MD, FCAP ; editors, Stephen B. Edge, MD, FACS [and 16 others] ; Donna M. Gress, RHIT, CTR-Technical editor ; Laura R. Meyer, CAPM-Managing editor. Chicago IL: American Joint Committee on Cancer, Springer; 2017.

3. Charlson ME, Pompei P, Ales KL, MacKenzie CR. A new method of classifying prognostic comorbidity in longitudinal studies: development and validation. J Chronic Dis. 1987;40:373–83.

4. Jensen VM, Rasmussen AW. Danish Education Registers. Scand J Public Health. 2011;39 7 Suppl:91–4.

5. Denmark - Employment, Social Affairs & Inclusion - European Commission. https://ec.europa.eu/social/main.jsp?catId=1107&langId=en&intPageId=4493. Accessed 16 Mar 2023.

6. Pedersen P, Aagesen M, Tang LH, Bruun NH, Zwisler A-D, Stapelfeldt CM. Risk of being granted disability pension among incident cancer patients before and after a structural pension reform: A Danish population-based, matched cohort study. Scand J Work Environ Health. 2020;46:382–91.

7. Mailand M. Dagpengereformer og flexicurity i forandring. Faos, Copenhagen University. 2015.
